# Flying ticks: anciently evolved associations that constitute a risk of infectious disease spread

**DOI:** 10.1186/s13071-015-1154-1

**Published:** 2015-10-15

**Authors:** José de la Fuente, Agustín Estrada-Peña, Alejandro Cabezas-Cruz, Ricardo Brey

**Affiliations:** SaBio. Instituto de Investigación en Recursos Cinegéticos IREC-CSIC-UCLM-JCCM, Ronda de Toledo s/n, 13005 Ciudad Real, Spain; Department of Veterinary Pathobiology, Center for Veterinary Health Sciences, Oklahoma State University, Stillwater, OK 74078 USA; Facultad de Veterinaria, Universidad de Zaragoza, 50013 Zaragoza, Spain; Center for Infection and Immunity of Lille (CIIL), INSERM U1019 – CNRS UMR 8204, Université Lille Nord de France, Institut Pasteur de Lille, 59019 Lille, France; Ricardo Brey Studio, Galglaan 13, B-9000 Gante, Belgium

**Keywords:** Tick, Bird, Evolution, Genetics, Pathogen

## Abstract

Ticks are important vectors of emerging zoonotic diseases affecting human and animal health worldwide. Ticks are often found on wild birds, which have been long recognized as a potential risk factor for dissemination of ticks and tick-borne pathogens (TBP), thus raising societal concerns and prompting research into their biology and ecology. To fully understand the role of birds in disseminating some ticks species and TBP, it is important to consider the evolutionary relationships between birds, ticks and transmitted pathogens. In this paper we reviewed the possible role of birds in the dissemination of TBP as a result of the evolution of host-tick-pathogen associations. Birds are central elements in the ecological networks of ticks, hosts and TBP. The study of host-tick-pathogen associations reveals a prominent role for birds in the dissemination of *Borrelia* spp. and *Anaplasma phagocytophilum*, with little contribution to the possible dissemination of other TBP. Birds have played a major role during tick evolution, which explains why they are by far the most important hosts supporting the ecological networks of ticks and several TBP. The immune response of birds to ticks and TBP has been largely overlooked. To implement effective measures for the control of tick-borne diseases, it is necessary to study bird-tick and bird-pathogen molecular interactions including the immune response of birds to tick infestation and pathogen infection.

**Electronic supplementary material:**

The online version of this article (doi:10.1186/s13071-015-1154-1) contains supplementary material, which is available to authorized users.

## Introduction

### Birds and risks of dissemination of tick-borne diseases

Vector-borne diseases are a growing problem for human and animal health worldwide [1]. Ticks are important vectors of emerging zoonotic diseases and both adult and immature stages are often found on wild birds [2]. Birds have been long recognized as a potential risk factor for dissemination of ticks and tick-borne diseases, thus raising societal concerns and prompting research into their biology and ecology. Birds can potentially transport tick-borne pathogens (TBP) that cause disease in humans and animals such as *A. phagocytophilum* (human and animal granulocytic anaplasmosis and tick-borne fever in ruminants), *Rickettsia* spp. (human and animal rickettsiosis), *Borrelia* spp. (human and animal borreliosis) by different means including transportation of ticks, infection with TBP and transmission to feeding ticks [3–10]. Therefore, although it has never been demonstrated that ticks or TBP have been established in a new locality after being transported by birds, evidence strongly suggests that this event may be possible [8].

To address the possible risks for dissemination of tick-borne infectious diseases by birds, it is important to understand the evolution of bird-tick-pathogen interactions and the ecologic and genetic drivers of these associations. Furthermore, understanding how birds respond to tick infestations and pathogen infection may provide new interventions for reducing the risks for spreading of ticks and transmitted pathogens by birds. Recent reviews have addressed some of these questions [2, 8, 11], but in this paper we reviewed the possible role of birds in the dissemination of tick-borne diseases as a result of the evolution of host-tick-pathogen associations.

To review the possible role of birds in the dissemination of tick-borne diseases as a result of the evolution of host-tick-pathogen associations, we focused as a model on bacterial pathogens of the genera *Borrelia*, *Rickettsia*, *Anaplasma*, *Ehrlichia* and *Neoehrlichia.* Viruses and protozoan pathogens and Relapsing fever *Borrelia* spp. were excluded from the analysis because certain tick-bird-pathogen associations such as those for *Babesia*, *Theileria* and viruses are difficult to support with current reports from the literature. Additionally, host-tick-pathogen networks were calculated from the compilation of published data spanning the period 1990–2014 by focusing as an example on taxonomic associations among these organisms in the western Palearctic only. The western Palearctic was defined as countries included within the borders marked by Scandinavia in the north, the Azores in the Atlantic, North African countries in the south, and the Ural Mountains and Turkey in the east. Therefore, some of the pathogens such as Relapsing fever *Borrelia* spp. were excluded from the analysis because they are poorly reported in the target region thus providing limited evidence for tick-host-pathogen association for these species. We explicitly excluded the records on domestic animals and the *Anaplasma* spp. such as *A. marginale* and *A. ovis* that primarily infest domestic animals, because it has been demonstrated that this data distorts the actual ecological structure underlying the “natural” network (see Additional file 1). Pathogen positive ticks and hosts considered in the analysis included both infected and pathogen DNA-positive records in published data.

## Review

### Historical perspective

The Ancient Egyptians and Greeks were aware of ticks. Tick fever is referred to in an Egyptian papyrus dated 1550 BC and in the Odyssey (850 BC) Homer wrote, “there lays Argos, the dog, full of dog flies” (kynoraistes, believed to be ticks) [12, 13]. In Egyptian hieroglyphs, “Sparrow”  was used as a determinative for “common” and “small” but also for “bad” [14] for birds becoming a pest but perhaps also for carrying ectoparasites such as ticks. Since then ticks have been recognized as dangerous for human and animal health.

Fossil ticks are difficult to find but the record supports tick-bird co-evolution [15]. Fossil ticks that have birds as possible hosts range from 90–94 Mya (Cretaceous) to 15–40 Mya (Tertiary) [15]. These species include *Carios jerseyi*, *Ixodes succineus*, *I. tertiarius*, *Amblyomma near testudinis*, unclassified *Ixodes*, *Hyalomma*, *Amblyomma* species, and *Ornithodoros antiquus* [15]. Interestingly, the oldest fossil corresponds to *C. jersey* (90–94 Mya) with the hypothesis that the tick fed on sea-faring birds to explain how it was found in New Jersey amber [16]. Recently, *Borrelia*-like spirochetes were found in fossil *Amblyomma* sp. in Dominican amber [17], providing the first record of spirochete-like cells associated with fossil ticks and providing additional support for the possible role of birds in disseminating this pathogen. Additionally, a tick discovered in prehistoric Arizona coprolite of human origin supports the hypothesis that ticks were a potential source of disease and that ancient people ate ectoparasites [18].

**Fig. 1:**
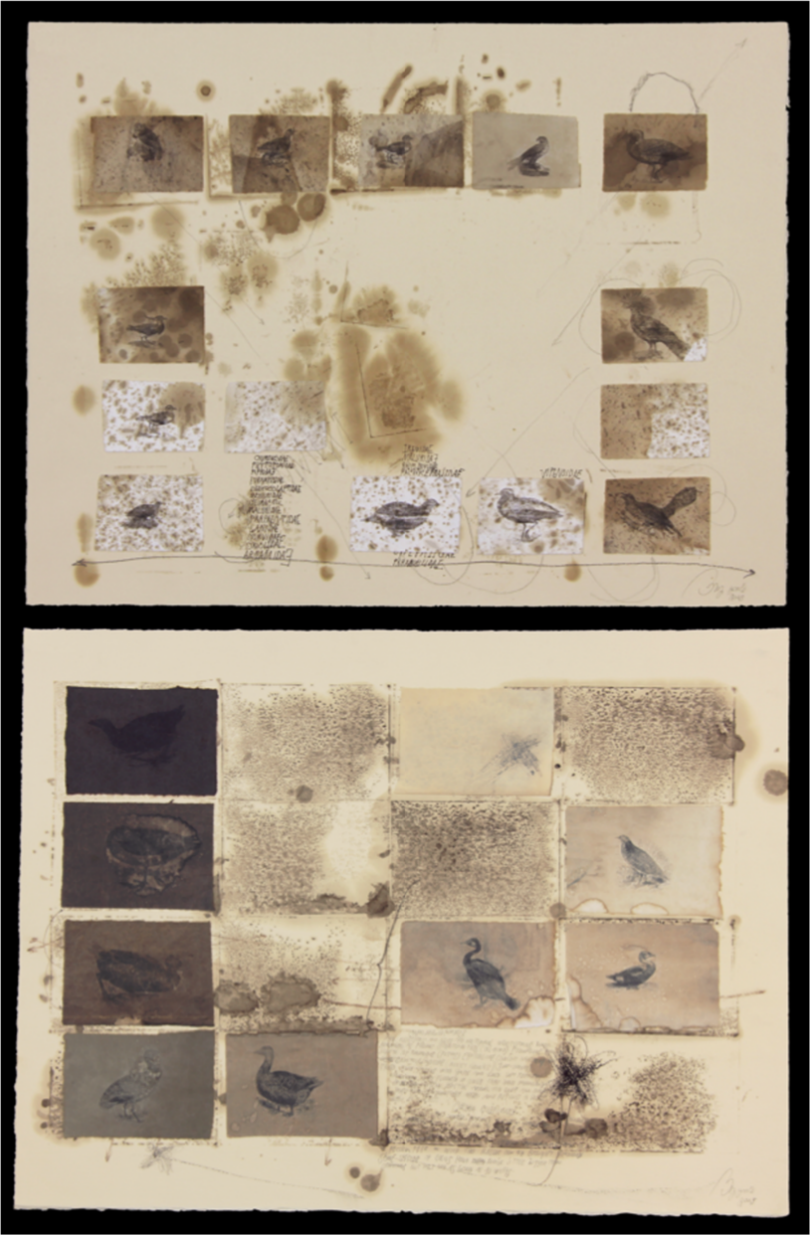
Artist interest in birds natural history. This piece by Ricardo Brey (http://www.ricardobrey.com) illustrates societal concerns about the risks associated with the possible role of birds in disseminating ectoparasites and infectious diseases. Form the series “Clados”. Mixed media on paper, 2009 (50 × 65 cm). Courtesy KGJ Collection, Spain. Photo: Isabel Brey

The role of birds in disseminating ectoparasites and infectious diseases have raised societal concerns and prompted interest into the study of their biology and ecology (Fig. 1). However, when the terms “tick” AND “bird” were used to search PubMed (http://www.ncbi.nlm.nih.gov/pubmed/) only 1343 publications were found and patents were not found on the World Intellectual Property Organization, the United States Patent and Trademark Office, the European Patent Office and the Free Patents Online databases (www.biowebspin.com/) on August 28, 2015. These findings indicated that despite the role of birds as a potential risk factor for dissemination of ticks and tick-borne diseases, more research is needed to better understand bird-tick-pathogen interactions and to develop strategies for the control of tick infestations and TBP in these species.

### Evolutionary considerations of bird-tick-pathogen associations

**Fig. 2:**
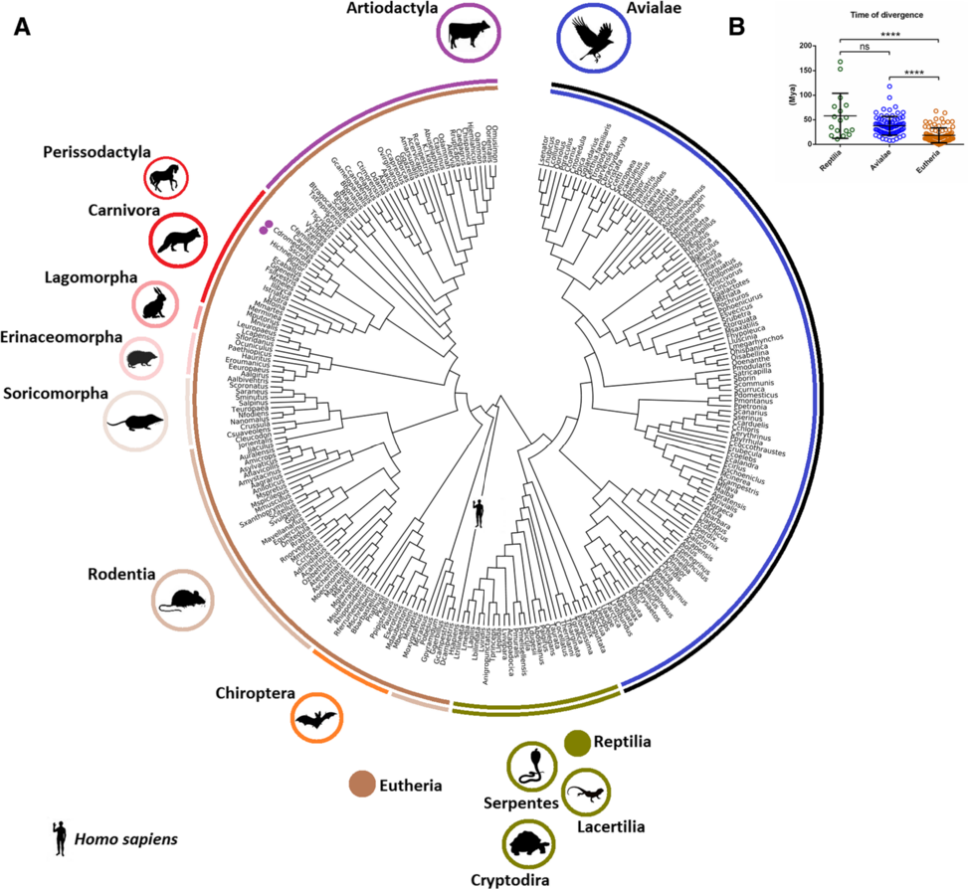
Diversity of vertebrate hosts for ticks and TBP. **a** Tree of the major vertebrate hosts for ticks and TBP was reconstructed using *cytochrome b*(*Cytb*) nucleotide sequences from 265 vertebrates (see Additional file 1). Amphibians were not included in the analysis because only one amphibian (*Bufo marinus*) is considered to be regularly infested by ticks, *Amblyomma rotundatum* [43]. **b** Divergence times for major hosts for ticks and TBP: Birds (Average 37.68 ± 19.08 Mya), Mammals (Average 18.27 ± 15.22 Mya) and Reptiles (Average 57.93 ± 45.85 Mya). Asterisks indicate levels of significance in the differences between groups tested by Kruskal-Wallis test post hoc Dunn’s multiple comparison test (****p < 0.0001; ns, no significant differences)

Birds are important components of the ecological networks between ticks, hosts and pathogens. The centrality of birds in the network of TBP transmission is consistent with the diversity of birds that host ticks and TBP (Fig. 2a) (see Additional file 1). Birds alone account for approx. 50 % of animals that host ticks and TBP.

The evolutionary relationships between birds, ticks and transmitted pathogens are important to understand the role of birds in disseminating ticks and TBP. Bird species that support ticks and TBP are older (37.68 ± 19.08 Mya) than Eutherian (mammals) (18.27 ± 15.22 Mya) species (Fig. 2b), suggesting that the evolutionary associations between ticks, pathogens and birds may precede that of ticks, pathogens and mammals.

**Fig. 3:**
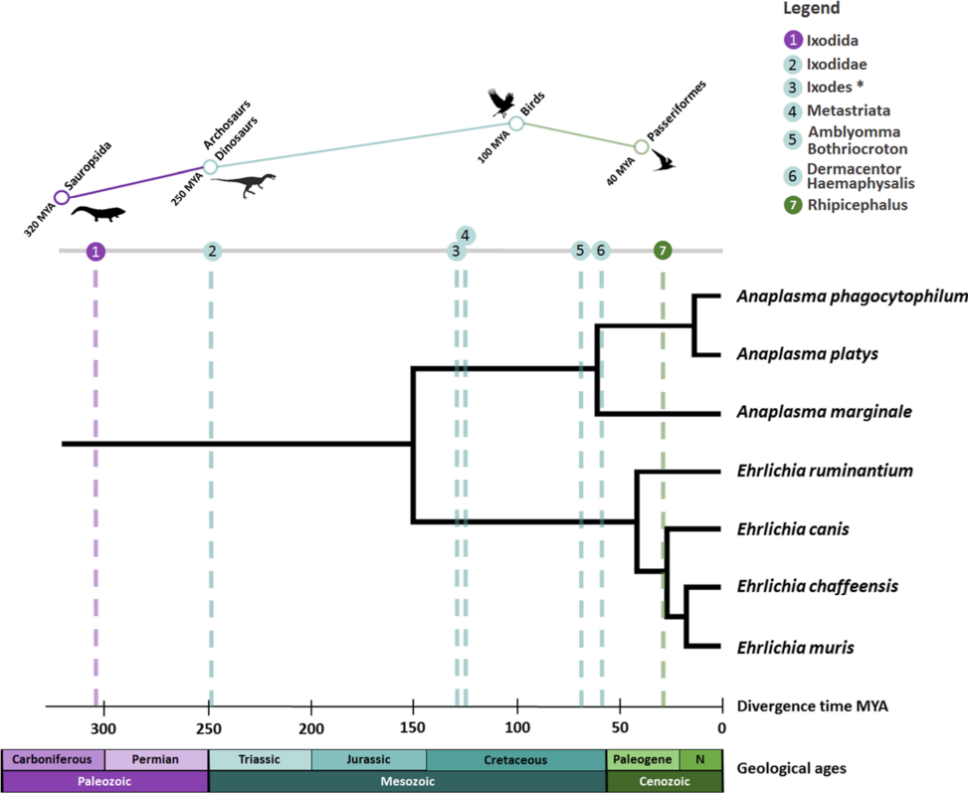
Estimated dates of divergence for ticks, *Anaplasma*, *Ehrlichia* and birds. The maximum likelihood tree of the genera *Anaplasma* and *Ehrlichia* was constructed using *16S rRNA* nucleotide sequences (see Additional file 1). Molecular clock analyses were performed for major tick lineages using CYTB amino acid sequences and for *Anaplasma* and *Ehrlichia* using *16S rRNA* nucleotide sequences (see Additional file 1). Divergence times of Sauropsida [44], Dinosaurs [21] and birds [24, 25], including Passeriformes birds [25], are shown. *Diversification of *Ixodes*, excluding Australian *Ixodes*. In geological ages line “N” is Neogene

To explore this hypothesis, we reconstructed and overlapped the history of the evolution of birds, mammals, ticks and TBP (Fig. 3). The molecular clock analysis positioned the origin of Ixodida in the late Carboniferous approx. 319 Mya [19]. This period was characterized by a rapid extinction of amphibian species after the rainforest collapse event 305 Mya [20]. This event also triggered an evolutionary burst among reptiles being ecologically adapted to the drier conditions that followed [20]. Thus, inadvertently, the rainforest collapse paved the way for the rise of dinosaurs, which started to diversify approx. 250 Mya [21], and is considered to be the ancestors of extant Archosaurs (group of amniotes whose living representatives consist of birds and crocodilians) [22–24] (Fig. 3). Mapping the main evolutionary diversifications between Ixodida showed that indeed they overlap with the major transitions in bird evolution (Fig. 3). The two main groups of hard ticks (Postriata and Metastriata) split approx. 250 Mya coinciding with the rise of Avemetatarsalia (group of Archosaurs that are closer to birds than to crocodiles) [21]. Non-Australian Ixodida and Metastriata split 124 Mya, coinciding with the split of Neognathae (a clade that includes virtually all living birds except the tinamous and the flightless ratites) and Palaeognathae (the clade that includes the tinamous and the flightless ratites) birds (approx. 100–110 Mya) [25]. The radiation of the tick genera *Amblyomma* (approx. 70 Mya), *Bothriocroton* (approx. 73 Mya), *Dermacentor* (60 Mya) and *Haemaphysalis* (approx. 62 Mya) concurred with the rapid radiation (within 10 to 15 Mya) of Neoaves (a clade that includes all modern birds with the exception of Paleognathae and Galloanserae) that took place approx. 65 Mya [25]. The diversification of *Rhipicephalus* (approx. 28.56 Mya) concurred with the point at which the major Passeriformes were established approx. 25 Mya [25].

Despite radiations of non-Australian Ixodida and Metastriata, Neognathae and Palaeognathae birds and mammals (Rodentia, Carnivora and Artiodactyla) concurred approx. 100 Mya, it is interesting to note that the divergence of Rodentia (approx. 91.8 Mya), Carnivora (approx. 84.9 Mya) and Artiodactyla (approx. 87.3 Mya) [26], which are important components of extant tick-host networks (Fig. 2a), occurred before the radiation of abundant tick genera (5, 6 and 7 in Fig. 3). However, as mentioned before, molecular clock analysis of divergence times for host species showed that mammals (including Rodentia, Carnivora and Artiodactyla) to which ticks and transmitted pathogens are associated with are relatively younger than birds (*p* < 0.0001) (Fig. 2b). Altogether, these facts suggest that tick-bird associations are provably older than tick-mammal associations.

Regarding TBP, the results suggest that the association between ticks and birds are probably recent when compared to that of ticks and TBP (i.e. family Anaplasmataceae). The common ancestor of *Rickettsia* was a free-living bacterium that adapted to intracellular endosymbiosis with protists approx. 525–775 Mya [27, 28]. The transition to infecting arthropods occurred approx. 425–525 Mya, around the Cambrian explosion, when most metazoan phyla appeared [27]. Our molecular clock analysis is consistent with this hypothesis about the origin of *Rickettsia* and placed the divergence of *Anaplasma* and *Ehrlichia* approx. 150 Mya (Fig. 3), which is consistent with the divergence of the ancestor of the *Rickettsia* group (including groups hydra, torix and rhizobius) [27]. Several *Rickettsia*, mainly of the Spotted Fever Group, are associated to and transmitted by ticks [29]. Interestingly, the radiation of *Rickettsia* approx. 50 Mya concurred with the radiation of *Anaplasma* (approx. 61.32 Mya), *Ehrlichia* (approx. 41.51 Mya) and that of tick genera *Amblyomma* (approx. 70 Mya), *Bothriocroton* (approx. 73 Mya), *Dermacentor* (approx. 60 Mya) and *Haemaphysalis* (approx. 62 Mya).

Taken together, these results support the hypothesis that bird-tick-pathogen associations evolved in the Cretaceous approx.125 Mya, thus suggesting that ticks adapted to feed on birds, the likely host for early *Anaplasma* and *Ehrlichia* species long before mammalian hosts appeared on Earth. Consequently, birds likely played an important role in the dissemination of ticks and TBP to mammals.

### Bird-tick-pathogen ecological and phylogenetic associations

**Fig. 4:**
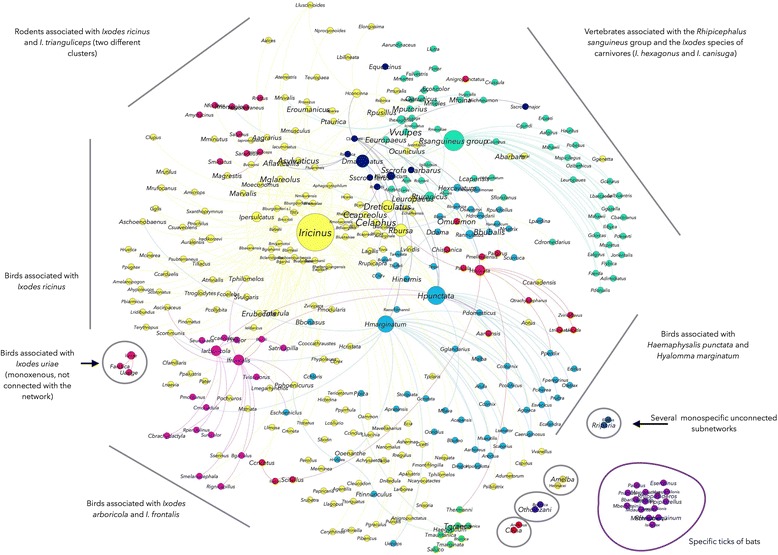
Network of ticks, vertebrate hosts (excluding domestic animals) and TBP. Networks were calculated from a compilation of published data spanning the period 1990–2014 on taxonomic associations among these organisms in the western Palearctic. A total of 15,342 records were summarized in 1022 unique different combinations of ticks, vertebrates and pathogens. The nodes and the edges of the networks were colored according to its modularity (see Additional file 1). For example, the nodes in yellow are the cluster of taxa represented by the tick *Ixodes ricinus*, three secondary tick species (*Rhipicephalus bursa*, *Dermacentor reticulatus* and *I. persulcatus*), their hosts, and the pathogens these ticks and vertebrates contribute to circulate. The size of each node is proportional to its Node Betweenness Centrality (NBC) and the size of the label is proportional to its PageRank (PR) (see Additional file 1)

Network analysis (see Additional file 1) illustrates the complex relationship and the high number of taxa involved in the resilience of tick-host-pathogen associations in nature [30] (Fig. 4). A total of 13 clusters were detected in the network. Six of these clusters are groups of monoxenous ticks of vertebrate hosts, which are thus separated from the rest of the network. The network has 322 unique associations of tick species or tick-transmitted pathogens in which the Phylum Aves is involved (Fig. 4). In comparison, the Rodentia and Artiodactyla, which are considered some of the most important tick hosts and reservoirs of TBP [31, 32], were recorded in only 176 and 115 unique associations, respectively. Other than for the few species of monoxenous ticks of vertebrate hosts (bat ticks *Ixodes simplex* and *I. vespertilionis, I. lividus*on *Riparia*, *Haemaphysalis erinacei*, *Argas reflexus*, *Ornithodoros tholozani* and *I. uriae*.), a complete segregation of the hosts and ticks into unique and closed groups was not found. Seven clusters are interconnected by several tick species, which are shared by various vertebrate hosts in the clusters (Fig. 4). Ticks are not ectoparasites of groups of phylogenetically related vertebrate hosts, but tend to be environmentally related with them [30]. Therefore, many species of vertebrates share tick species from different clusters because they share the same environmental niche and not because of a particular preference of the tick for a group of vertebrate hosts.

**Fig. 5:**
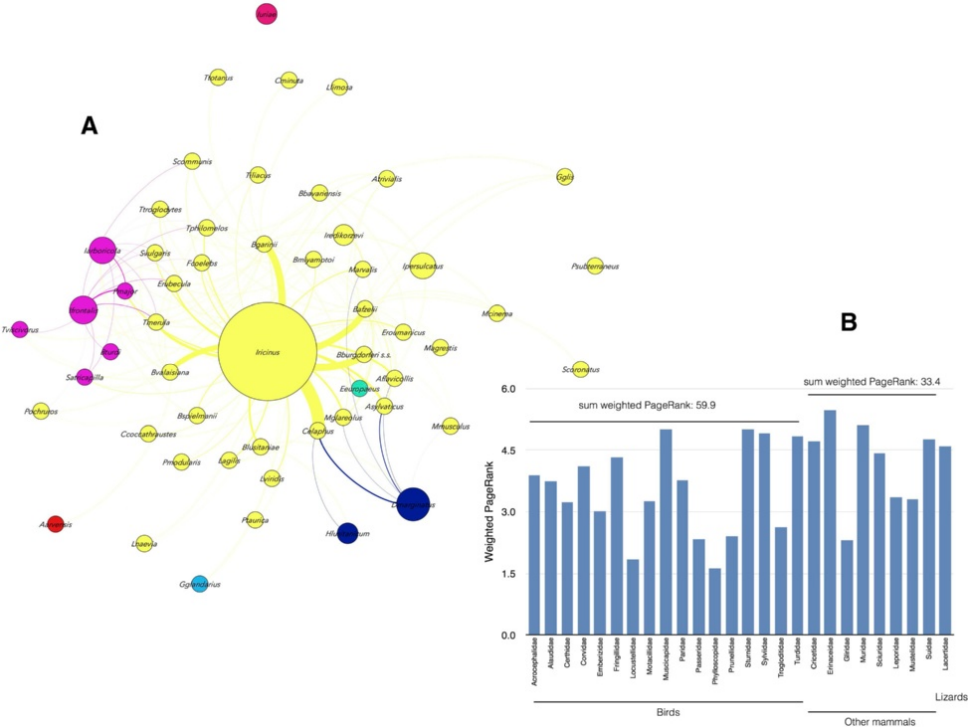
Host-*Borrelia*-tick associations. **a** Network of vertebrate hosts, tick vectors and *Borrelia* spp. The original coloring and symbol sizes of Fig. 3 were retained to show the involvement of taxa from different clusters in the resilience of *Borrelia* spp. in nature. **b** Comparison of the weighted PR (see Additional file 1) between families of birds, mammals and reptiles to analyze the relative contribution of vertebrate hosts to the circulation of *Borrelia* spp. in the network

Birds are hosts of 22 species of ticks and have been recorded as reservoirs for a total of 10 species of TBP of the genera *Anaplasma*, *Borrelia* and *Rickettsia.* The relationships of ticks and TBP of the genera *Borrelia*, *Rickettsia*, *Anaplasma*, *Ehrlichia* and *Neoehrlichia* with birds in the network was explored to capture the ecological and phylogenetic signatures of the clusters in the network and to understand how birds can contribute to support the circulation of these pathogens. It is well known that bacteria of the genus *Borrelia* tend to segregate according to the phylogenetic group of the reservoir host [33]. Mammals, reptiles and birds support different *Borrelia* spp., all of them linked to the group of ticks belonging to the cluster of *I. ricinus* [34]. The genus *Borrelia* circulates through a heterogeneous assemblage of ticks and reservoir hosts (Fig. 5a). Reservoir hosts include 17 families of birds, 8 families of mammals and 1 family of reptiles from 7 different clusters of the network. Most prominent in this cluster are the rodents and birds associated with *I. ricinus* and ticks from other clusters such as *I. frontalis* and *I. arboricola*. Ticks and birds of the *I. frontalis* group play a prominent role in the circulation of *Borrelia* spp. and the results support the hypothesis that the contribution of birds to the resilience of *Borrelia* spp. in nature is almost twice that of rodents (Fig. 5b). This is a novel result because although *Borrelia* spp. are phylogenetically associated with groups of certain reservoir hosts [33], network analysis suggested that the relevance of each vertebrate host in supporting the circulation of ticks is different.

**Fig. 6:**
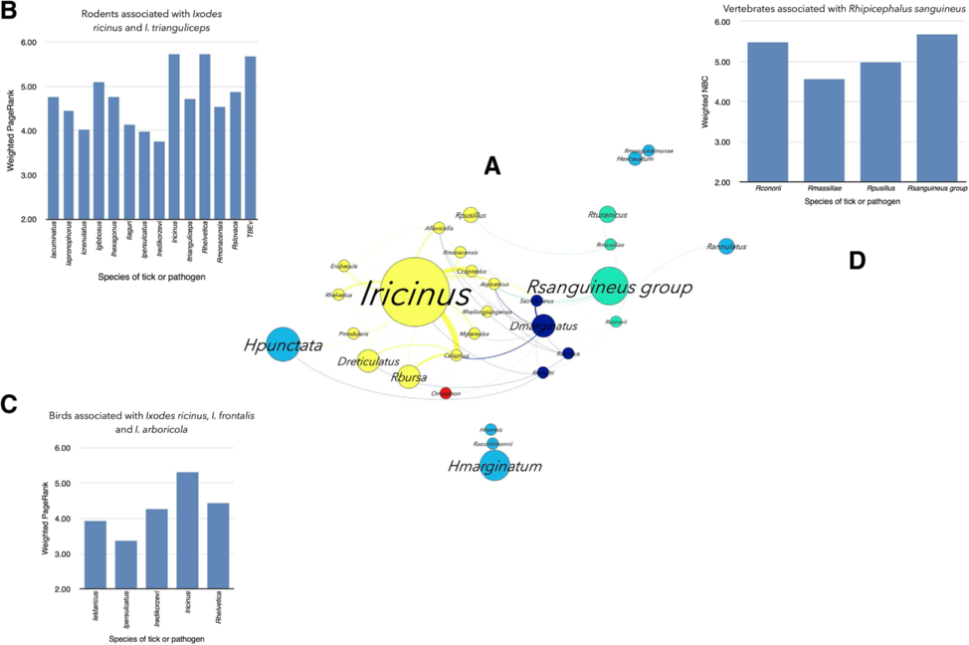
Host-*Rickettsia*-tick associations. **a** Network of vertebrate hosts, tick vectors and *Rickettsia* spp. The original coloring and symbol sizes of Fig. 3 were retained to show the involvement of taxa from different clusters in the resilience of *Rickettsia* spp. in nature. **b** Values of PageRank for the different species of ticks and pathogens included in this cluster, associated to the rodents that are hosts of *I. ricinus* or *I. trianguliceps*. **c** Values of PageRank for the different species of ticks and pathogens included in this cluster, associated to the birds that are hosts of *I. ricinus*, *I.frontalis* or *I. arboricola*. **d** Values of PageRank for the different species of ticks and pathogens included in this cluster, associated to the vertebrates that are hosts of the *R. sanguineus* group

The rickettsiae of the genera *Rickettsia*, *Ehrlichia*, *Anaplasma* and *Neoehrlichia* are a group of pathogens that circulate between many species of ticks and vertebrate hosts [35]. *Anaplasma marginale* and *A. ovis* were not considered because they primarily infest domestic animals that were excluded from the analysis. The pathogens of the genus *Rickettsia* are widely segregated among the clusters of the network (Fig. 6a). *Rickettsia slovaca*, *R. heilonjgianjensis* and *R. monacensis* are restricted to rodents related to the cluster of *I. ricinus*. However, *R.helvetica* circulates between both rodents and birds associated with *I. ricinus*, and with the birds associated to *I. frontalis* and *I. arboricola.* Therefore, in terms of associations, rodents support a higher variety of tick vectors therefore having a higher reservoir potential for *Rickettsia* spp. (Fig. 6b and c). *Rickettsia raoulti* and *R. slovaca* are associated to the tick *D. marginatus*, whose immature stages parasitize rodents. Other *Rickettsia* spp. such as *R. mongolotimonae* and *R. aeschlimanii* have been recorded only in the ticks *Hyalomma excavatum* and *Hyalomma marginatum* or *Haemaphysalis inermis*, respectively. Therefore, the different role of the vertebrates in the circulation of these two rickettsiae is currently unknown. There is yet another interesting finding regarding the rickettsiae of the species *R. conorii* and *R. massiliae*. While they have been reported on domestic carnivores, records from wild animals suggest that they have been recorded only in ticks of the genus *Rhipicephalus* (Fig. 6d)*.* In summary, the organisms of the genus *Rickettsia* have a well defined association with different clusters of either ticks or vertebrates. Birds are poor contributors to the circulation of these pathogens except for *R. helvetica*. One hypothesis to explain these results is that these organisms have an ecological association to these clusters, i.e. *Rickettsia* occupies different ecological niches following the environmental preferences of the ticks, therefore “filling” different niches as a result of speciation. The alternative hypothesis is that *Rickettsia* are phylogenetically tied to their reservoirs, therefore segregating into different clusters because of phylogenetic relationships between reservoir hosts. The first hypothesis is well supported by our data because birds in which no other *Rickettsia* but *R. helvetica* have been recorded, share only two species of ticks with rodents, in which four species of *Rickettsia* have been recorded. All *Ixodes* spp. prefer humid and cold environments, which are clearly different to the steppe habitats characteristics of *Hyalomma* spp. and of the Mediterranean habitat preferred by *Rhipicephalus* spp. In fact, the radiation of rickettsiae around 50 Mya concurred with radiation of vertebrate and tick species other than *Ixodes* spp.

**Fig. 7:**
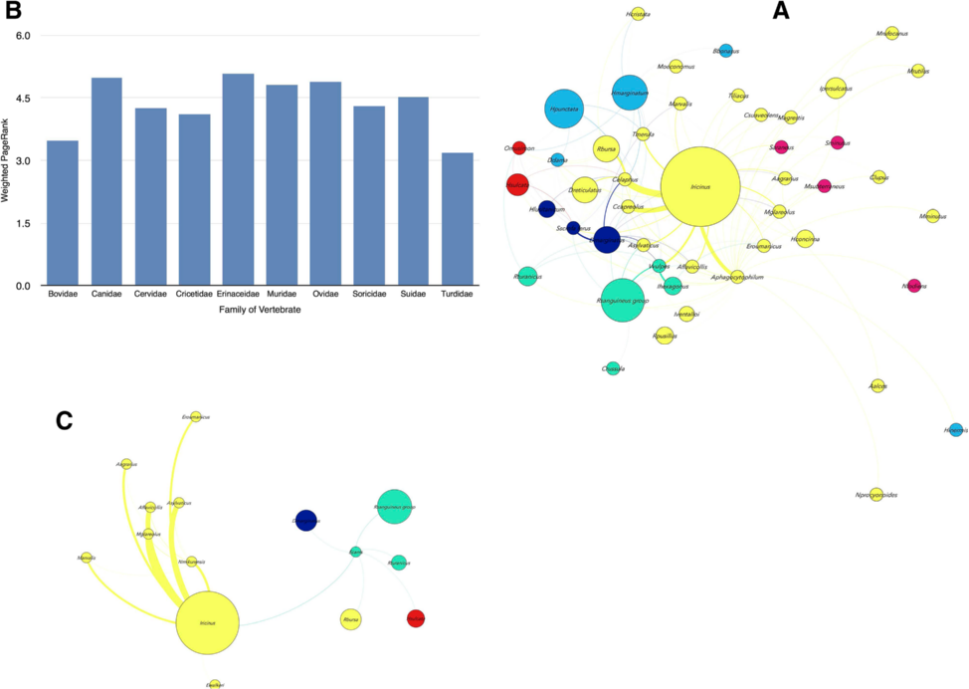
Host-*A. phagocytophilum*-tick and host-*Neoehrlichia-E. canis*-tick associations. **a** Network of vertebrate hosts, tick vectors and *A. phagocytophilum*-tick, *Neoehrlichia* and *E. canis*. The original coloring and symbol sizes of Fig. 3 were retained to show the involvement of taxa from different clusters in the resilience of *A. phagocytophilum* in nature. **b** Values of PageRank for the different species of vertebrates included in this sub-network, supporting the circulation of Values of PageRank for the different species of ticks and pathogens included in this cluster. **c** The network of vertebrates and ticks involved in the sub-network. The original coloring and size of symbols of Fig. 3 were retained to show the involvement of taxa from different clusters in the resilience of *E. canis* and *N. mikurensis* in nature

The case of *Anaplasma phagocytophilum* is different (Fig. 7a). This rickettsia circulates through a large number of ticks and vertebrates in 7 clusters of the network. Up to ten families of reservoir hosts have been recorded for this rickettsia, of which the members of the family Turdidae are the only birds involved in its circulation. However, it is interesting to note that birds have a prominent contribution to the circulation of *A. phagocytophilum* in nature (Fig. 7b) which is similar to the reservoir potential of Bovidae, classically considered as some of the most prominent reservoirs of this rickettsia in nature [36]. It is not clear if strains of the pathogen are tied to specific groups of tick and/or vertebrate hosts. Recent evidence suggests that molecular data support the circulation of different strains of *A. phagocytophilum* in nature [37]. Whether these strains are phylogenetically tied to specific clusters of the network remains unsolved because lack of data for this bacterium. A similar situation exists in the case of *Ehrlichia canis,* which is tied to several tick species in four different clusters but has never been reported in wild animals. However, other *Ehrlichia* spp. such as *E. walkeri* and *Neoehrlichia mikurensis* are linked to only one cluster of ticks and reservoir hosts (Rodentia and Insectivora).

In summary, the study of host-tick-pathogen associations revealed a prominent role for birds in the dissemination of *Borrelia* spp. and *A. phagocytophilum*, with little contribution to the possible dissemination of other rickettsiae.

### Bird response to tick infestations and pathogen infection and possibilities for control of vector-borne diseases

Little information is available on the avian immune response to tick infestations and pathogen infection, which are a fundamental component of host-tick-pathogen interactions [2]. Ectoparasite-specific antibody response and non-specific antibody titers positively correlate with tick infestations in chicken and sand martin, respectively [2]. Furthermore, although selection on birds has favored a variety of possible adaptations for dealing with ectoparasites [11], genetic traits associated with tick resistance in birds have not been defined [2]. It has been suggested that the prevalence of ticks on different bird species depends mainly on the degree of feeding on the ground [8]. However, Clayton et al. [2] provide a number of adaptation mechanisms by which birds combat ectoparasite infestations. Recently, Benson et al. [38] demonstrated links between the predominantly extinct deep time adaptive radiation of non-avian dinosaurs and the phenomenal diversification of birds, via continuing rapid rates of evolution along the phylogenetic stem lineage. Furthermore, recent analyses revealed that pan-avian genomic diversity covaries with adaptations to different lifestyles and convergent evolution of traits [39]. Some of these mechanisms may be related to the adaptation to combat tick infestations and selection of genetic traits for tick resistance.

These results suggest that it is necessary to characterize bird response to tick infestations and pathogen infection using Next Generation Sequence (immunogenomics, transcriptomics, proteomics, and other omics) technologies and bioinformatics to identify genetic markers and mechanisms associated with tick infestations. As shown in other host species, these results together with the characterization of bird response to vaccination with ectoparasite-derived antigens may result in new interventions to control tick infestations and pathogen infection in birds [40–42], thus reducing the risks for spreading ticks and TBP.

## Conclusions

Birds are central elements in the ecological networks of ticks, hosts and TBP. To fully understand the role of birds in disseminating some ticks species and TBP, it is important to consider the evolutionary relationships between birds, ticks and transmitted pathogens. The study of host-tick-pathogen associations reveals a prominent role for birds in the dissemination of *Borrelia* spp. and *A. phagocytophilum*, with little contribution to the possible dissemination of other TBP. The implementation of effective measures to control tick-borne diseases is associated with the understanding of ecological factors affecting the dynamics of TBP transmission and biological mechanisms such as immune responses resulting from the interaction between ticks, reservoir hosts and pathogens. The immune response of birds to ticks and TBP has been largely overlooked. Birds have played a major role during tick evolution, which explains why they are by far the most important hosts supporting the ecological networks of ticks and several TBP. To implement effective measures for the control of tick-borne diseases, it is necessary to study bird-tick and bird-pathogen molecular interactions including the immune response of birds to tick infestation and pathogen infection.

## Additional file

Additional file 1:
**Methods.** Description of methods used for network, phylogenetic and molecular clock analyses. (DOCX 124 kb)
