# Supplementary material for: Flying ticks: anciently evolved associations that constitute a risk of infectious disease spread
Source: Parasit Vectors. 2015 Oct 15;8:538. doi: 10.1186/s13071-015-1154-1 (PMC4607018; doi:10.1186/s13071-015-1154-1)
Supplement: Additional file 1: — Methods. Description of methods used for network, phylogenetic and molecular clock analyses. (DOCX 124 kb) [file 13071_2015_1154_MOESM1_ESM.docx]

**Additional file 1. Methods.**

Description of methods used for network, phylogenetic and molecular clock analyses.

**Network analysis**

Networks represent system components (nodes) and the relations between those components (links). Each node represents a species, and the resulting link between two nodes represents a relationship. In the same way that food webs are descriptions of who eats whom in an ecosystem, the network in Figures 3-6 are a description of who is a parasite of whom, and who is a carrier of whom regarding tick-transmitted pathogens, their vectors and reservoir hosts. Consequently, the network is directed, i.e. each edge links a pathogen to a vertebrate or a vector. Data on pairs of systematic associations among ticks and vertebrates, pathogens on vertebrates, and pathogens on ticks were compiled from a literature review focused on the western Palearctic, which was defined as countries included within the borders marked by Scandinavia in the north, the Azores in the Atlantic, North African countries in the south, and the Ural Mountains and Turkey in the east. We explicitly excluded the records on domestic animals, because it has been demonstrated that this data distorts the actual ecological structure underlying the “natural” network (Estrada-Peña et al., 2015). A “record” is a combination of pathogens/ticks/vertebrates at one site, that we call herein “partners”. These combinations are always dyadic, involving a pathogen detected in a tick, a pathogen detected in a vertebrate, or a tick collected on a vertebrate. To represent the network, each node symbolizes a species, and the resulting edge between two nodes represents a relationship, for example “pathogen A detected in tick B,” “pathogen A detected in vertebrate C,” or “tick B recorded on vertebrate C.” The literature review was completed in December 2014. A total of 15,342 records of pairs of ticks and vertebrates, TBP and ticks, or TBP and vertebrates were assembled and converted into a network structure for further analysis.

We first computed the modularity of the network. Modularity computes sets of nodes that are more connected among them than with other nodes in the network. The result is a picture of clusters of species that while still connected to members of other clusters, are more closely linked to the members of its own cluster. The network is not only a graphical representation, but the relationships among the partners can be used to track important ecological indexes that quantify the properties of the network. Measures of centrality were produced to understand the specific contribution of each partner to the network coherence. The Node Betweenness Centrality (NBC) and the PageRank (PR) of each node are measures of the importance of each node (taxa) in the network. The NBC is defined as the number of times a node is the shortest path between two any other nodes. In our application, NBC measures the importance of a particular node for the circulation of the parasitic and infectious agents in the network. The PR assigns a universal rank to nodes based on the importance of the other nodes to which it is linked. A node has a high PR if the sum of the ranks of the organisms linked to that node is high. For example, a tick may have a high PR if it transmits prominent pathogens and feeds on vertebrates that are well connected in the network. The PR index thus measures the importance of a node in the network, not only because the node links a high number of species of other organisms, but also due to the relative importance of the organisms linked. NBC and PR provide complementary information.

Host-parasite data are sensitive to sampling effort. Consequently, the computation of individual centralities is largely influenced by the intensity of sampling and reporting. To ensure that our findings are robust, we used an approach employed in similar studies of controlling for variation in sampling effort (Gómez et al., 2013). Specifically, we regressed the weight of each edge against the number of citations of the least sampled species (vertebrate, tick, TBP) in each edge. Afterwards, we additively rescaled the residuals to be greater than zero. The residuals would reflect the number of links relative to sampling effort, under the assumption that the measure of sampling effort should be from the less studied species. We replaced the original weights of the edges (number of parasites shared per pair of vertebrate species) by the rescaled residuals, and then computed all the centrality estimates.

**Phylogenetic and molecular clock analyses**

The phylogenetic tree of the order Rickettsiales, including family Anaplasmataceae, was reconstructed using *16S rRNA* nucleotide sequences as reported previously (Weinert et al., 2009). Nucleotide sequences were collected from Pelagibacter (GenBank accession number K2S160), *Holospora obtuse* (X58198), *Anaplasma marginale* (M60313), *A. phagocytophilum* (M73224), *A. platys* (EF139459), *Ehrlichia ruminantium* (X61659), *E. canis* (GU810149), *E. chaffeensis* (NR_074500), *E. muris* (NR_121714); three Rickettsia from the “hydra” group which were isolated from *Hydra oligactis* (EF667896), *Carteria cerasiformis* (AB688628) and *Pleodorina japonica* (AB688629); two *Rickettsia* from the “torix” group which were isolated from *Torix tagoi* (AB066351) and *Torix tukubana* (AB113214); *Rickettsia felis* (NR_074483), *R. Helvetica* L36212, *R. japonica* (NR_074459), *R. prowazekii* (NR_044656), *R. conorii* (NR_074480), *R. bellii* (CP000849), *R. rickettsia* (CP000848); *Candidatus Midichloria* (NR_074492) and *Orientia tsutsugamushi* (AM494475) was used to root the tree. The phylogeny of ticks was reconstructed with Cytochrome b (CYTB) amino acid sequences from members of all major tick families: *Ixodes persulcatus* (NP_739605), *I. hexagonus* (NP_008508), *I. holocyclus* (NP_945238); *Rhipicephalus microplus* (AGH19718), *R. sanguineus* (NP_008522), *R. annulatus* (AGH19675); *Amblyomma americanum* (ABA19090), *A. fimbriatum* (YP_006234442), *A. triguttatum* (YP_044790), *A. cajennense* (YP_007475027); *Haemaphysalis flava* (NP_945226), *H. formosensis* (YP_007475040), *H. hystricis* (AFU55303), *H. humerosa* (AFU55312); *Argas africolumbae* (YP_007026379), *A. miniatus* (YP_009000449), *A. lagenoplastis* (YP_009000423), *Ornithodoros moubata* (NP_722573), *O. porcinus* (NP_996550), *O. brasiliensis* (YP_009000475); *Bothriocroton concolor* (YP_006234403), *B. undatum* (YP_006234416) and *Dermacentor silvarum* (AJK90817). Four species of Mesostigmata were used to root the tree of ticks: *Metaseiulus occidentalis* (YP_001095994), *Stylochyrus rarior* (YP_003288848), *Phytoseiulus persimilis* (YP_003587337) and *Varroa destructor* (NP_758884).

The groups of sequences were aligned separately with MAFFT (v7) configured to maximize accuracy (Katoh and Standley, 2013). After alignment, regions with gaps were removed using Gblocks configured for relaxed selection of blocks (Castresana, 2000; Talavera and Castresana, 2007). A total of 230 and 1391 gap free positions resulted from the alignment of bacteria and tick sequences, respectively. The model of sequence evolution having the lowest Bayesian Information Criterion (BIC) scores for each gene was used. The models were identified using MEGA6 (Tamura et al., 2013). The evolutionary models used were as follows: *16S rRNA* – GTR+G and CYTB – GTR+G+I. The phylogenetic trees were reconstructed using the maximum likelihood method implemented in MEGA 6 (Tamura et al., 2013). The reliability of internal branches was tested using 1000 bootstrap replications.

To date the divergence of the major clades of *Rickettsia* and ticks, timetrees were built using RelTime implemented in MEGA6 (Tamura et al., 2013). The *16S rRNA* tree was calibrated setting the divergence of Orientia and hydra at 225 Mya (Weinert et al., 2009). The CYTB tree of ticks was calibrated setting the divergence of Argasinae and Ornithodorinae (234 Mya), Ixodida and Mesostigmata (350 Mya), Ixodida (319 Mya), Ixodidae (249 Mya), Metastriata (124 Mya) and *Ixodes* (217 Mya) following Mans et al. (2012). The tree of vertebrate hosts was reconstructed using *Cytb* nucleotide sequences from 265 vertebrates. To calibrate the vertebrate host tree, divergence times obtained in TimeTree (Hedges et al., 2006; Kumar and Hedges, 2011) were used:

| **Taxon A** | **Taxon B** | **Divergence (Mya)** |
| --- | --- | --- |
| **Eutheria** | | |
| *Giraffa camelopardalis* | *Ovis aries* | 29.4 |
| *Ovis aries* | *Tragelaphus spekii* | 30.9 |
| *Ovis aries* | *[Canis lupus familiaris](http://www.ncbi.nlm.nih.gov/Taxonomy/Browser/wwwtax.cgi?mode=Undef&name=Canis%20lupus%20familiaris&lvl=0&srchmode=1)* | 84.6 |
| *Ovis aries* | *Atelerix albiventris* | 91.4 |
| **Bats** | | |
| *Rhinolophus hipposideros* | *Myotis blythii oxygnathus* | 61.3 |
| **Rodents** | | |
| *Mus musculus* | *Rattus rattus* | 25.4 |
| *Meriones crassus* | *Dipodillus campestris* | 7.6 |
| *Mus musculus* | *Jaculus jaculus* | 66.0 |
| **Bats/Rodents** | | |
| *Rhinolophus hipposideros* | *Jaculus jaculus* | 94.2 |
| **Reptiles** | | |
| *Natrix natrix* | *Testudo graeca* | 230.7 |
| **Aves-Reptiles** | | |
| *Natrix natrix* | Alectoris graeca | 274.9 |
| **Aves** | | |
| *Alectoris graeca* | *Turdus pilaris* | 100.9 |

The statistical significance between the divergence times of species within Reptilia, Avialae and Eutheria was evaluated using the non-parametric Kruskal–Wallis test in the GraphPad 6 Prism program (GraphPad Software Inc.).

**References**

Castresana, J. (2000). Selection of conserved blocks from multiple alignments for their use in phylogenetic analysis. Molecular Biology and Evolution 17, 540-552.

Estrada-Peña, A., de la Fuente, J., Ostfeld, R.S., Cabezas-Cruz, A. 2015. Interactions between tick and transmitted pathogens evolved to minimise competition through nested and coherent networks. Scientific Reports 5: 10361.

Gómez, J. M., Nunn, C. L., & Verdú, M. (2013). Centrality in primate–parasite networks reveals the potential for the transmission of emerging infectious diseases to humans. *Proceedings of the National Academy of Sciences*, *110*(19), 7738-7741.

Hedges SB, Dudley J & Kumar S (2006) TimeTree: a public knowledge-base of divergence times among organisms. Bioinformatics 22:2971-2972.

Katoh, K., & Standley, D. MAFFT multiple sequence alignment software version 7: improvements in performance and usability. Mol. Biol. Evol. 30, 772-780 (2013).

Kumar S and Hedges SB (2011) TimeTree2: species divergence times on the iPhone. Bioinformatics 27:2023-2024.

Talavera, G., and Castresana, J. (2007). Improvement of phylogenies after removing divergent and ambiguously aligned blocks from protein sequence alignments. Systematic Biology 56, 564-577.

Tamura K, Stecher G, Peterson D, Filipski A, Kumar S (2013) MEGA6: Molecular Evolutionary Genetics Analysis version 6.0. Molecular Biology and Evolution:30 2725-2729.

Weinert LA, Werren JH, Aebi A, Stone GN, Jiggins FM. Evolution and diversity of Rickettsia bacteria. BMC Biol. 2009 Feb 2;7:6.
